# The Hidden Culprit: A Computed Tomography Diagnosis of H-Type Tracheoesophageal Fistula in an Adult Revealed After Two Decades of Pulmonary Infections

**DOI:** 10.7759/cureus.70983

**Published:** 2024-10-07

**Authors:** Mounir Salek, Soufiane Bigi, Hajar Arache, Manal El Moujahid, Soukaina Wakrim

**Affiliations:** 1 Radiology, Souss Massa University Hospital, Agadir, MAR

**Keywords:** bronchiectasis, case report, ct scan, respiratory infection, trachea oesophageal fistula

## Abstract

We report a case of a 41-year-old male with undocumented cardiopathy, managed with furosemide, spironolactone, digoxin, and acetylsalicylic acid, presenting with progressive dyspnea and recurrent respiratory infections since childhood. The patient exhibited worsening dyspnea over four months, escalating to New York Heart Association (NYHA) classification stage IV. Physical examination revealed lower limb edema, right pleural effusion, jugular vein distension, hepatojugular reflux, bilateral crepitant rales, and abdominal distension. Diagnostic workup showed right axis deviation on ECG, bilateral bronchial and interstitial syndrome on chest X-ray, and negative GeneXpert (Cepheid, Sunnyvale, California, United States) for Koch's bacillus. Transthoracic echocardiography indicated paradoxical septum and right cavity dilation. Computed tomography (CT) scan revealed bilateral cystic bronchiectasis, right pleural effusion, mediastinal and hilar lymphadenopathies, right cardiac chambers dilation, tracheal diverticulitis, tracheoesophageal communications, and a left main bronchus-esophagus fistula. Laboratory tests indicated leukocytosis with neutrophilia. The patient was admitted to the Intensive care unit (ICU) for respiratory infection management, pulmonary artery hypertension control, and bypass of the tracheoesophageal fistula (TEF) with a nasogastric tube pending surgical repair. This case highlights a probable congenital H-type TEF as the underlying cause of the patient's recurrent respiratory infections. TEF, typically diagnosed at birth, can rarely present in adulthood, with the H-type being a rare form. Symptoms often include recurrent pneumonia, hemoptysis, and meal-associated coughing. Diagnosis involves thoracic imaging and endoscopy, with esophagography and CT scans being critical for detecting the fistula. Treatment options include surgical and endoscopic interventions, with the cervical approach often preferred for operative correction. Early diagnosis of congenital TEF is crucial for preventing recurrent infections and associated complications. Healthcare providers should maintain vigilance for TEF in patients with unexplained recurrent respiratory infections, ensuring timely and effective management through appropriate diagnostic and therapeutic strategies.

## Introduction

This article presents a 41-year-old male with undocumented cardiopathy and recurrent respiratory infections, ultimately diagnosed with a congenital H-type tracheoesophageal fistula (TEF). Despite TEF typically being identified at birth, this case highlights its potential to manifest in adulthood, emphasizing the need for a thorough investigation of persistent respiratory symptoms.

## Case presentation

A 41-year-old male with a history of undocumented cardiopathy, currently managed with furosemide, spironolactone, digoxin, and acetylsalicylic acid, presented with recurrent respiratory infections since childhood, with the first hospitalization at age 20.

The patient reported progressive dyspnea over the past four months, initially classified as New York Heart Association classification (NYHA) stage II and progressing to NYHA stage IV within the past week. He also reported a productive cough and a sensation of fever, while maintaining a preserved general state.

Clinical examination revealed edema of the lower limbs, right pleural effusion syndrome, jugular vein distension with hepatojugular reflux, bilateral crepitant rales, and a distended abdomen.

Investigations showed an ECG with right axis deviation, and a chest X-ray revealing bilateral bronchial syndrome, bilateral interstitial syndrome, and right pleural syndrome. The GeneXpert (Cepheid, Sunnyvale, California, United States) test for Koch's bacillus was negative. Transthoracic echocardiography showed a paradoxical septum with dilation of the right cavities.

Laboratory findings included a white blood count of 13,140/mm³ with neutrophils at 9,030/mm³, lymphocytes at 4,075/mm³, and eosinophils at 35/mm³.

**Figure 1:**
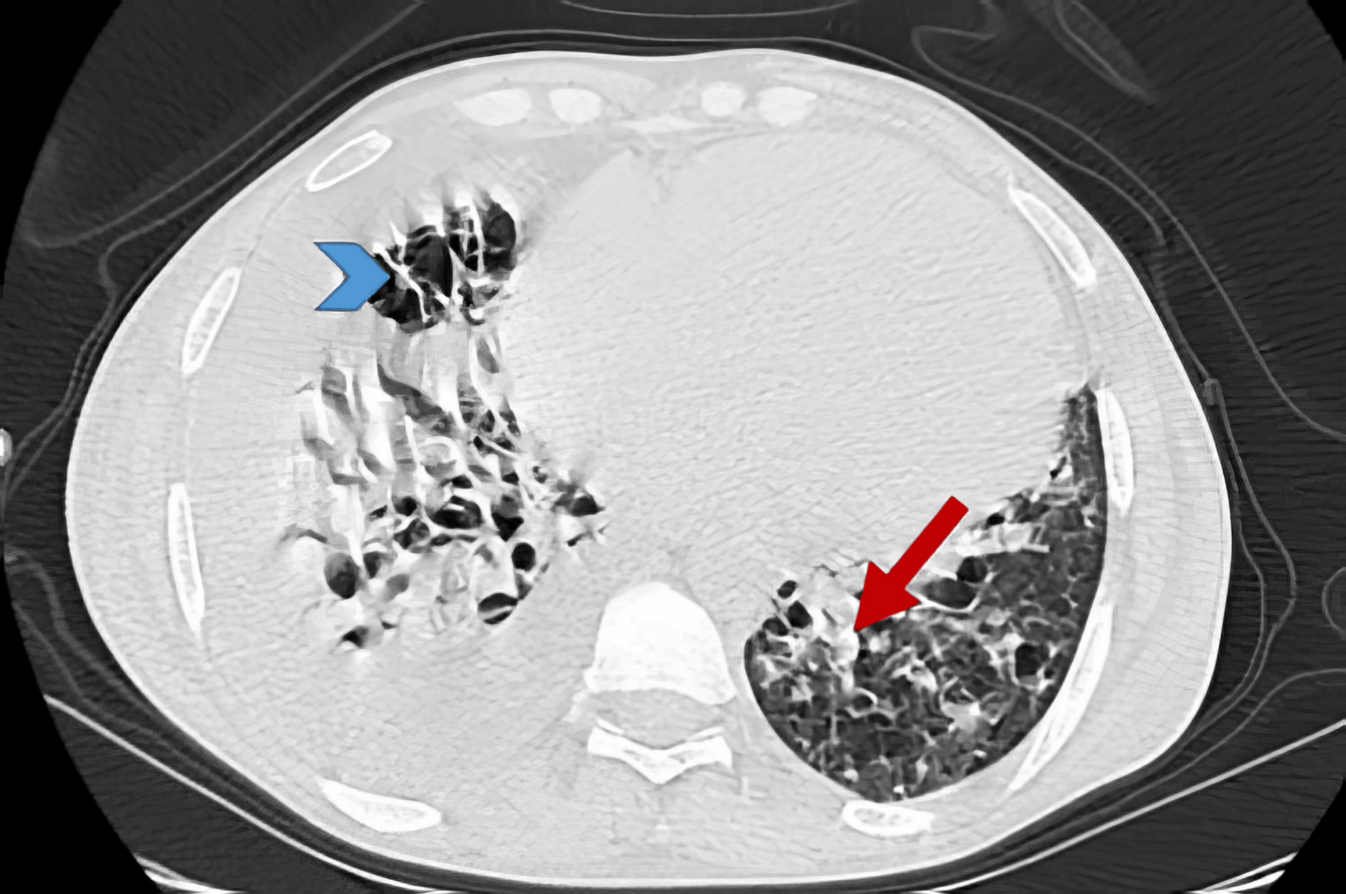
Axial CT scan image in lung window shows bilateral cystic bronchiectasis (blue arrow head) predominantly in the lower lobes with mucoid impactions (red arrow).

**Figure 2:**
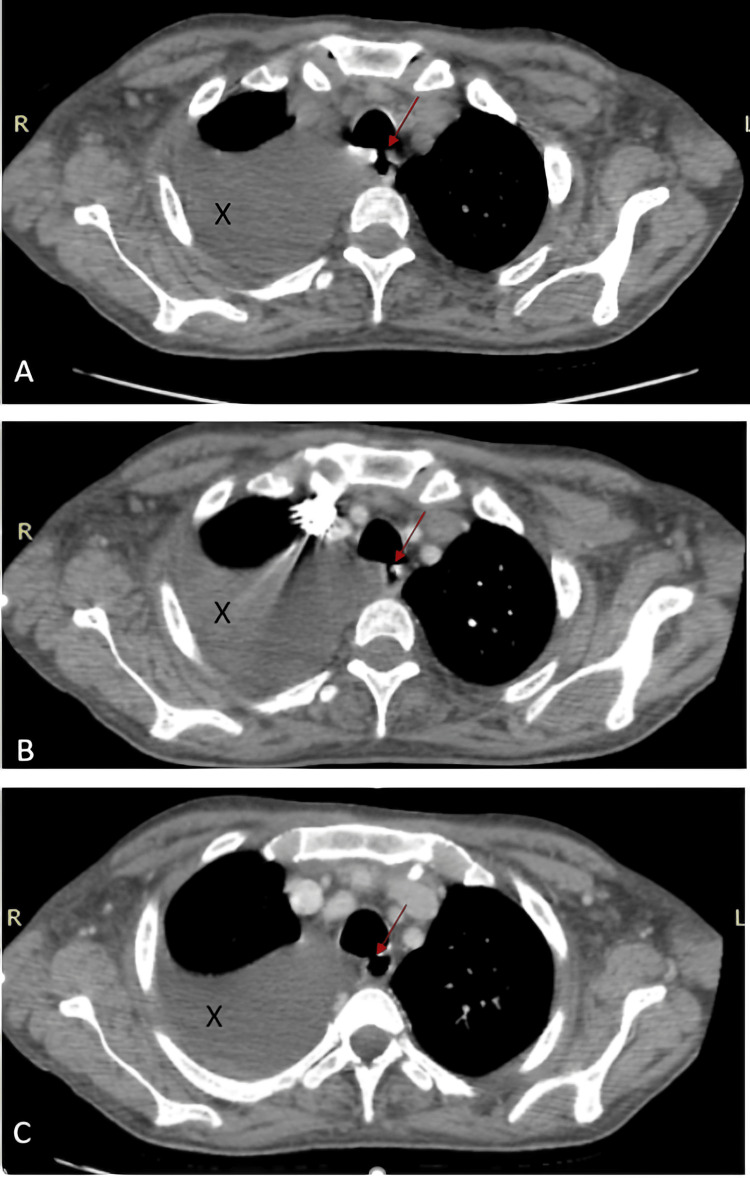
Axial CT scan images without contrast (A), in arterial phase (B), and venous phase (C) showing the TEF (red arrow) and pleural effusion (black X). TEF: tracheoesophageal fistula

CT scan showed bilateral cystic bronchiectasis predominantly in the lower lobes with mucoid impactions (Figure 1), a moderate right pleural effusion (Figure 2), and multiple mediastinal and left hilar lymphadenopathies, with the largest at station 11L.

**Figure 3:**
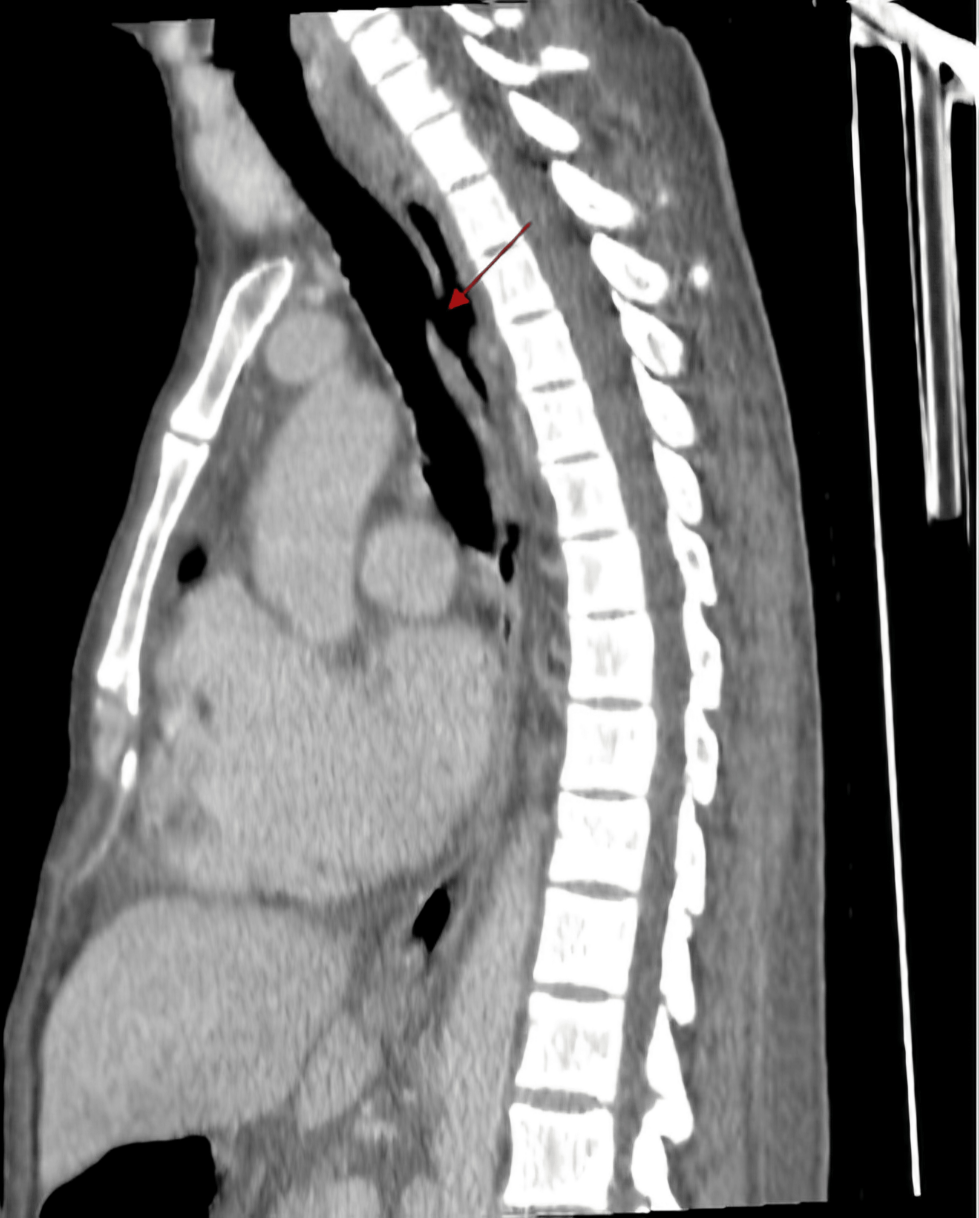
Sagittal CT scan image with contrast showing the H-type trachea esophageal fistula (red arrow).

There was dilation of the right cardiac chambers with a right ventricle/left ventricle (RV/LV) ratio >1, tracheal diverticulitis, tracheoesophageal communications at the level of T3 and T4 vertebrae visualized on different acquisition in time, and a fistula between the left main bronchus and the esophagus (Figure 3).

The patient was admitted to the intensive care unit for management, aiming to treat his respiratory infection, control his pulmonary artery hypertension, and bypass his TEF with a nasogastric tube while awaiting surgical repair of the fistulas.

## Discussion

In this article, we present the case of a 41-year-old male with undocumented cardiopathy and recurrent respiratory infections since childhood. The underlying cause was identified as a probable congenital H-type TEF. This case highlights the importance of thoroughly investigating recurrent respiratory infections.

Congenital TEF, commonly recognized in infancy, can also manifest later in adulthood. It can occur as an isolated condition or in association with esophageal atresia. In our case, we found no other evident congenital malformation associated. Congenital TEF occurs in approximately 1 in 4,100 individuals [1]. Although typically diagnosed at birth, TEF can rarely present in adults, with the literature on this subject being limited to case reports and small case series. However, the H-type TEF, which does not involve esophageal atresia, may go undiagnosed until adulthood. This type is the rarest form of TEF, accounting for only 4% of all congenital cases, with an incidence of approximately one in 100,000 live births [2]. Common symptoms include recurrent pneumonia, hemoptysis, and coughing during meals. Early diagnosis and treatment are essential to prevent repeated infections, chronic lung damage, inflammation, and potential pulmonary sepsis. Seventy percent of H-type TEFs are located at or above the level of the second thoracic vertebra. They typically follow an oblique path resembling the letter N, extending from the posterior membranous wall of the trachea to the anterior wall of the esophagus. Symptoms of H-type TEF typically begin at birth and may include choking, coughing, and cyanosis due to aspiration of food, excessive tracheal secretions, and bubbly respiration (often described by caretakers as a "mucousy" child). Recurrent respiratory infections, symptom improvement with gastric tube feeding, and abdominal distention caused by aerophagia are key indicators that should alert clinicians to the possibility of an H-type fistula.

TEF diagnosis involves thoracic imaging and endoscopy, including flexible bronchoscopy and upper endoscopy if feasible. Initially, respiratory symptoms are evaluated with a chest radiograph, which can show various changes depending on symptom duration, ranging from bibasilar infiltrates to more defined basilar consolidative changes. While formal guidelines are lacking, esophagography and endoscopy are usually used for both diagnosing the disease and planning for surgery [3]. Esophagography is typically performed using barium due to its safer profile compared to gastrograffin, which has been associated with pulmonary edema and death due to its hypertonic nature [4]. During esophagography, the contrast will pass through the fistula and be visible in the airways, aiding in the visualization of the defect. This method can detect the defect in approximately 70% of patients with TEF [5]. For patients who cannot swallow contrast, such as those who are sedated or ventilated, a chest CT scan may be performed to evaluate for signs of fistula, aerodigestive tract anatomy, and mediastinal pathology, although the diagnostic performance of CT scans in TEF is not well-established.

In the majority of cases, CT scans reveal a direct connection between the esophagus and the trachea. Additionally, several indirect findings aid in TEF detection, such as thickening of the esophageal or airway walls, fatty stranding in the mediastinum, distention of the esophagus with air, presence of fluid or debris within the airways, and lung abnormalities indicative of aspiration or infectious pneumonia.

The treatment of H-type TEF can vary between surgical treatment, endoscopic treatment, or coupling surgery with endoscopic investigation. Limited data exist on the endoscopic treatment of congenital H-type TEFs. Bhatnagar et al. reported the use of electrocautery and neodymium-doped yttrium aluminum garnet laser (Nd:YAG laser) for vaporizing the mucosal lining in one patient for the treatment of TEFs [6]. In a separate study, Lelonge et al. reported successful outcomes in 14 cases of TEF treated with rigid bronchoscopic chemo-cauterization using trichloroacetic acid (TCA) [7]. Operative correction for TEF involves two main approaches, lateral cervical or thoracic. The accurate estimation of the fistula level is crucial for selecting the appropriate approach. While the thoracic approach was initially favored, it has been found to be challenging due to the high location of the fistula [8]. In contrast, the cervical approach is often more suitable and preferred in most cases [9]. Before the operation, it is recommended to couple surgery with endoscopic investigation [10]. This involves using guide wires and catheters for cannulation, which aids in the division of the fistula during surgery.

## Conclusions

In conclusion, early diagnosis of congenital tracheoesophageal fistula (TEF) is paramount. Given its association with recurrent pulmonary infections, prompt recognition and intervention can significantly improve patient outcomes. Healthcare providers should maintain a high index of suspicion for TEF in infants and children presenting with respiratory symptoms, particularly recurrent infections. Further investigation, including imaging studies and endoscopic evaluation, is crucial for confirming the diagnosis and guiding appropriate management. Early identification of congenital TEF not only prevents complications but also ensures timely and effective treatment, highlighting the importance of vigilance and proactive diagnostic strategies in clinical practice.
